# Rare Presentation of Herpes Virus Lesions in a Case of Acute Pre-B Lymphoblastic Leukemia

**DOI:** 10.4274/tjh.galenos.2018.2018.0372

**Published:** 2020-02-20

**Authors:** Eylem Kaymaz, Zeliha Güzelküçük, Melek Işık, Neşe Yaralı

**Affiliations:** 1 University of Health Sciences, Ankara Child Health and Diseases Hematology Oncology Training and Research Hospital, Clinic of Pediatrics, Ankara, Turkey

**Keywords:** Herpes virus, Leukemia, Child

## To the Editor,

**Figure 1:**
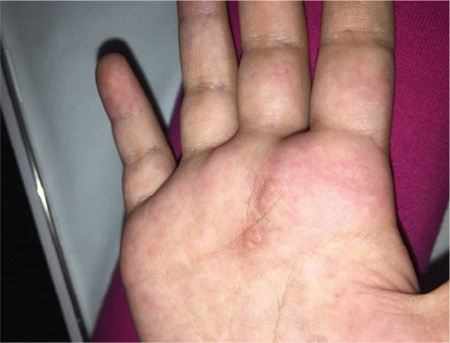
Vesicular lesions located on the hyperemic skin of the palm.

A 6-year-old girl with the diagnosis of acute pre-B lymphoblastic leukemia had febrile neutropenia and pneumonia after induction chemotherapy. Though wide-spectrum antibiotics were started and then antifungal treatment was added, the fever could not be controlled. During this period, a small vesicle resembling herpes labialis developed at the edge of her lip and acyclovir was added. The patient’s respiratory distress improved with combined antibacterial and antifungal therapy and saturation increased to normal levels after 1 week. During this period when the patient was afebrile, 3-5 vesicles were noted on her palm (Figure 1).

Herpes simplex virus (HSV) has two types, HSV-1 and HSV-2, and these viruses are members of the herpesviruses family. HSV can usually have lesions on different areas of body. Clinical presentations range from asymptomatic infection to mucocutaneous infections such as orolabial, ocular, genital herpes, herpetic whitlow, herpes gladiatorum, and eczema herpeticum as well as neonatal herpes, herpetic encephalitis, and fatal dissemination [1,2]. The diagnosis of HSV infection can mostly be done with the clinical appearance of the lesions and the history of the patient. It mostly produces oral and perioral lesions but it may disseminate systematically and cause secondary bacterial and fungal infections [3]. In children, HSV infections on the hand most commonly occur on the fingers and thumb, called herpetic whitlow. This infection can be secondary to autoinoculation of the virus from a primary oral HSV infection such as gingivostomatitis or inoculation by a different person who bites or sucks on the finger [4,5]. The palmar area is involved less commonly and can be transmitted to others through contact with skin vesicles and also in patient skin-to-skin contact. In our patient, the palmar lesion was transmitted from her labial herpes.
